# Precision Medicine Through Network Language: Integrating Clinical Insight and Data Expertise

**DOI:** 10.3390/genes17040467

**Published:** 2026-04-16

**Authors:** Maria Concetta Palumbo, Lorenzo Farina, Manuela Petti

**Affiliations:** 1 Institute for Applied Computing (IAC) “Mauro Picone”, National Research Council of Italy, Via dei Taurini 19, 00185 Rome, Italy; c.palumbo@iac.cnr.it; 2 Department of Computer, Control and Information Engineering “A. Ruberti”, Sapienza University of Rome, Via Ariosto 25, 00185 Rome, Italy; manuela.petti@uniroma1.it

**Keywords:** network-based precision medicine, interdisciplinary collaboration, data integration, clinical–bioinformatics interface, molecular tumor boards, disease modules, translational bioinformatics, high-dimensional data interpretation

## Abstract

Precision medicine is facing a critical transition driven by the growing complexity of biological data and the insufficient ability of current models to translate such data into clinically meaningful information. Linear, single-gene approaches are no longer adequate to explain the multifactorial nature of most modern diseases, whose phenotypes emerge from combinations of genetic, molecular, and environmental factors. Network-based precision medicine addresses this by providing a systemic framework capable of integrating heterogeneous omics data, interactomes, and clinical information to identify disease modules and novel therapeutic opportunities. The distinct novelty of this review is its focus on the potential of “network language” as the primary driver for realizing precision medicine through professional collaboration. We argue that networks are not merely tools that achieve precision “per se”; rather, their transformative power lies in their ability to serve as a shared and interpretable interface grounded in network theory. By offering this common conceptual ground, the paradigm bridges the deep cultural and methodological gaps between clinicians and data analysts, enabling effective cooperation between figures with fundamentally different, and often divergent, backgrounds. Practical tools—such as biological network analysis and Molecular Tumor Boards—demonstrate how computational modeling and clinical expertise can be successfully combined to generate actionable insights. Ultimately, network-based precision medicine represents a decisive step toward reconstructing the patient’s complexity and promoting a genuinely personalized clinical approach in which quantitative analysis and medical reasoning act synergistically through multidisciplinary integration.

## 1. Introduction

Precision medicine promises “the right treatment to the right person, at the right time” [1]. This idea contrasts with the “traditional” version in the sense that traditional medicine defines therapy based on the disease a patient has, rather than on the patient who has the disease. The strength of precision medicine lies in the fact that it considers individual differences based on each patient’s gene sequences, lifestyle, and environment. Its greatest success has certainly been the emergence of a new class of “molecularly targeted” drugs, in which the specific mutations observed in the patient are targeted. We can no longer ignore the evidence that new diagnostic and therapeutic paradigms have become a vital necessity. Despite the high expectations generated in the last two decades, the clinical impact of precision medicine has so far proved to be limited and uneven, with benefits often restricted to small subgroups of patients. The great difficulty in finding reliable diagnostic biomarkers and new effective treatments for complex and long-standing diseases, such as cancer, diabetes, myocardial infarction, or Alzheimer’s disease, is now widely documented [2,3]. This is also due to the high biological heterogeneity and poor reproducibility of many preclinical results [4,5]. In oncology and neurology in particular, the high failure rate in the development of new drugs and the difficulty of translating molecular discoveries into real clinical improvements have fueled the perception of a conceptual and therapeutic stalemate [6,7].

The innovative concept proposed in this work is to view the “network” approach in precision medicine not primarily as a mathematical or formal tool capable, in itself, of generating more accurate predictions. Instead, the central thesis is that the true value of network-based strategies lies in their capacity to serve as a common language, enabling communication and integration between traditionally siloed domains of expertise—specifically between the clinician and the big data analyst [8]. In this perspective, the network concept is presented not as a mere computational method but as a shared conceptual framework. This framework allows professionals with divergent backgrounds, objectives, and reasoning modalities to collaborate more effectively. The clinician contributes essential knowledge regarding biological mechanisms, the pathophysiological context, and the clinical relevance of observed phenomena; meanwhile, the data expert provides the skills necessary to manage, integrate, and analyze vast quantities of complex information. The language of networks offers a representative structure capable of making explicit the intricate relationships between biological, clinical, and molecular elements, thereby facilitating productive dialogue between these two perspectives [9,10,11,12].

The central aim of this work is not to provide an exhaustive survey of existing clinical applications, but rather to articulate and clarify the role of network-based precision medicine as a shared conceptual and operational language capable of fostering effective collaboration between clinicians and data scientists. This review is intended as a foundational and perspective-driven contribution, designed to highlight a critical yet often underappreciated prerequisite for successful translation into practice: the establishment of a common framework that enables meaningful interdisciplinary interaction. More specifically, we seek to raise awareness among both clinical and data-oriented communities of the potential of network-based representations to bridge disciplinary boundaries. By emphasizing this integrative function, we aim to encourage the development of genuinely collaborative research efforts, including the design of future clinical trials and the implementation of network-informed strategies in healthcare settings. We argue that without such a shared understanding and without recognizing networks as tools for collaboration, rather than solely as abstract or predictive models, the advancement of precision medicine will remain limited. Therefore, the focus is intentionally placed on stimulating this cultural and methodological shift, which we consider a necessary condition for the emergence of robust clinical applications.

To our knowledge, this is one of the first reviews to explicitly explore the potential of the network concept through a linguistic lens and interdisciplinary collaboration [8]. Innovation, therefore, does not reside in the mathematical tool itself, but in the new mode of interaction between clinicians and data analysts. This synergy opens the door to richer, more contextualized interpretations of biomedical information, leading to a deeper understanding of data and, consequently, new opportunities for the advancement of precision medicine [12,13].

## 2. Current Status of Precision Medicine

Awareness of the frightening immensity and depth of the sea of life and its manifestations is very important to fully understand that in the ocean of complexity of Nature, there are (almost) never simple solutions or linear explanations [14,15]. The basic mindset when dealing with biological or medical problems must be one of extreme intellectual humility, always keeping in mind that the fundamental characteristic of life is its diversity [16]. As every living being is unique, every patient is unique and should be studied as such [17]. The process resembles that of an investigative path where, step by step, one must collect evidence, select the relevant evidence, look for new evidence and, in the end, build a credible, understandable and evidence-supported story that can explain what happened and why [18]. This increasing emphasis on individualized and evidence-driven approaches, however, also reflects a broader challenge within modern medicine: translating complex biological variability into effective therapeutic solutions.

**Figure 1:**
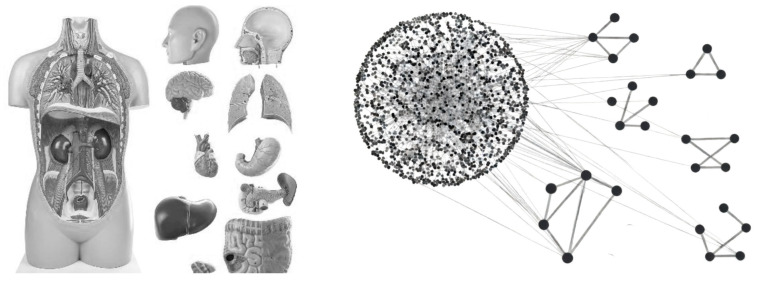
Extreme data fragmentation complicates patient-specific reconstruction, as networks can split into nested submodules.

In this context, the development of new drugs is steadily declining while the cost per unit is growing exponentially [19], putting a new pharmaceutical product on the market today costs more than one billion euros. Every day, millions of people take ineffective drugs [17]. One paper also states that, if we consider the ten best-selling drugs in the US, we find that they improve—on average—the condition of one in eleven patients. For example, rosuvastatin, commonly used to lower blood cholesterol levels, helps improve the condition of one in twenty patients [17]. Precision medicine, while promising a revolution in personalization, faces insurmountable obstacles that prevent the identification of new integrated diagnostic markers and truly effective and specific therapies. Despite the availability of large amounts and varieties of omics data, the transition to a truly personalized clinical practice is hampered by inherent biological complexities, methodological limitations, and the ever-increasing fragmentation of knowledge that prevents the patient from being reassembled after “taking him/her apart” at the molecular level [3,20,21], as illustrated in Figure 1).

### 2.1. The Complexity of the Genotype–Phenotype Relationship

One of the main challenges of contemporary medicine is represented by the intrinsically multifactorial nature of most human diseases and chronic ones, including cancers, diabetes, autoimmune and cardiovascular diseases. Monogenic diseases, i.e., caused by a single mutation, are relatively rare and occur only in a small proportion of clinical cases [22,23]. On the contrary, multifactorial diseases are polygenic and occur because of dynamic interactions with many genetic variants, each with modest effects that fit into a complex context formed by environmental, behavioral and epigenetic factors [24,25]. This inherent biological complexity makes it impossible to identify clear direct causal relationships and requires, instead, the use of integrative and multidisciplinary approaches [26,27].

A single gene rarely has a direct effect; instead, it can play a well-defined role within a context composed of multiple simultaneous phenotypic traits through the combined effects of complex regulatory networks at different molecular levels. Furthermore, the effect of a mutation is neither fixed in time nor universal: it varies as a function of interaction with a multiplicity of genes, metabolic pathways and the specific cellular and physiological environment of a patient [28]. This phenomenon, known as “pleiotropy” and “epistasis”, demonstrates the extreme complexity of the relationship between genotype and phenotype that can incorporate nonlinear reciprocal effects [29,30].

Pleiotropy and epistasis represent two key concepts in the genetics of complex diseases. Pleiotropy occurs when a single genetic variant influences multiple distinct phenotypic traits, whereas epistasis refers to interactions among different genes, whereby the effect of one gene depends on the presence or absence of others. A well-documented example is cystic fibrosis [31]. Mutations in the *CFTR* gene exhibit pleiotropic effects, as a single genetic alteration can lead to multiple clinical manifestations, including pulmonary dysfunction, pancreatic insufficiency, and abnormalities of the reproductive system. At the same time, the phenotypic variability observed among patients cannot be explained solely by *CFTR* mutations; it also reflects epistatic interactions with other modifier genes that can influence disease severity, particularly in relation to inflammatory responses and susceptibility to infections. This example highlights how the combination of pleiotropic and epistatic effects contributes substantially to clinical complexity, underscoring the importance of considering genetic networks rather than single genes in the study of inherited disorders.

As a result, the often-publicized simplistic idea of “one gene–one disease” is in fact scientifically inadequate in the context of omics–big data medicine and multifactorial diseases [26,27]. It is easy to see an increasingly evident disproportion between the speed with which sequencing technologies allow us to identify new genomic variants and our actual inability to interpret their biological and clinical roles [9,32]. Thousands of mutations are catalogued every year, but only a minority are associated with known molecular functions or clearly defined pathological phenotypes [33].

### 2.2. The Decline in Pharmacological Efficiency (Eroom’s Law)

Paradoxically, despite extraordinary advances in biomedical, computational and experimental technologies, the overall efficiency of pharmaceutical research and development processes has undergone a marked and systematic decline. This phenomenon is well described by the so-called Eroom’s Law, the inverse of Moore’s Law, according to which the number of new drugs approved for every billion dollars invested was reduced by about 80 times between 1950 and 2010 [6,19]. In other words, the exponential increase in technological power and biological knowledge has not translated into a proportional increase in therapeutic output but rather has coincided with a worsening of the economic and scientific efficiency of the sector. One of the most interesting indicators is the very high failure rate of drug candidates along the clinical pipeline. It is estimated that about 90% of molecules that enter the clinical trial phase never reach final approval [6,7]. The main causes of failure are unexpected toxicity and lack of clinical efficacy, which often emerge only in the late stages of human trials, when the economic and time costs are already extremely high. This data highlights profound limitations in the preclinical models currently used to predict drug behavior in complex biological systems.

One of the fundamental reasons for such failures lies in the reductionist approach that has historically guided drug discovery. Most drug discovery strategies are based on the identification and modulation of a single molecular target, typically a protein considered causally responsible for a given pathology. However, real biological systems do not function as linear chains of cause and effect but as highly interconnected networks of genes, proteins, metabolites, and cellular signals. Intervening on a single node of this network can generate indirect, compensatory or emergent effects that are not predictable in simplified models [26,27]. As a result, a drug designed to target a specific target may be ineffective because the biological system activates alternative compensation pathways, or it may induce severe side effects due to interference with other apparently unrelated physiological processes. This problem is particularly relevant in complex and multifactorial diseases, such as cancer, neurodegenerative and cardiovascular diseases, in which the pathogenesis emerges from the interaction of multiple molecular components distributed on different biological scales [17,34].

In this context, the difficulties of pharmaceutical innovation cannot be interpreted as a simple technological failure but rather as a conceptual misalignment between the complexity of biological systems and the theoretical models used to study them. Overcoming the limitations imposed by Eroom’s Law requires a paradigm shift towards systems biology and network-based precision medicine approaches, in which drugs are no longer evaluated exclusively based on their affinity for a single target but based on their global impact on biological networks.

### 2.3. Incompleteness and Bias of Molecular Maps

**Figure 2:**
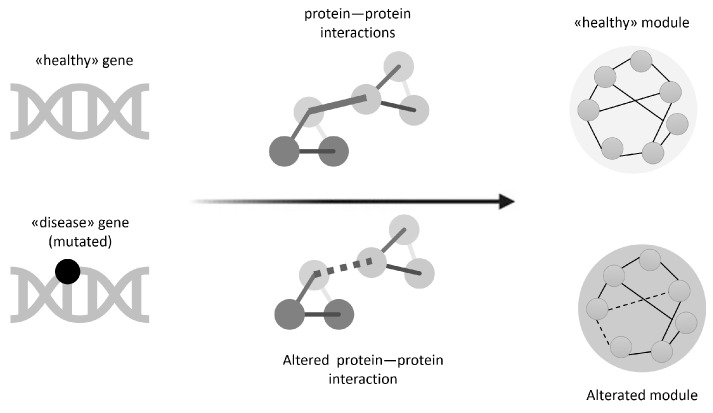
Schematic representation of the impact of a gene mutation on protein–protein interaction networks and module organization. In the healthy condition (**top**), a gene encodes proteins that engage in stable protein–protein interactions, forming a coherent functional module. In the disease state (**bottom**), a mutation in the gene alters specific protein–protein interactions (dashed lines), leading to a reorganization of the interaction network and disruption of the corresponding module structure.

Precision medicine relies heavily on the ability to map and interpret the molecular interactions that regulate the functioning of biological dynamical systems [35]. In this context, interactomes—the networks that describe the interactions between proteins, genes and other cellular components—represent a central conceptual and operational tool. However, such maps are still deeply incomplete, biased, and affected by experimental and historical biases. Current estimates indicate that the known human interactome covers less than 20% of the protein–protein interactions that exist [36]. This implies that most of the functional connections underlying physiological and pathological processes still remain unexplored. This incompleteness has direct consequences on the ability to identify and characterize the so-called “disease modules”, i.e., sets of proteins and genes that, when collectively perturbed, give rise to a specific pathological phenotype [26]. From a network-based precision medicine perspective, diseases are no longer considered the result of the alteration of a single gene but emerge from dysfunctions located in specific regions of the biological network, as illustrated in Figure 2. However, if the reference network is incomplete or biased, the identification of these modules is inevitably inaccurate, with the risk of identifying incomplete, secondary or even misleading targets.

A further structural problem is represented by the strong bias of research towards a limited number of genes and proteins already widely studied. Numerous analyses have shown that most experimental and clinical studies repeatedly focus on the same 10% or so of the human proteome, often referred to as the “illuminated proteome” [26]. This phenomenon is partly due to pragmatic factors, such as the availability of reagents, experimental models and previous data, which generates a vicious circle that strengthens the visibility of some nodes in the network to the detriment of others. As a result, a large portion of the human genome and proteome remains substantially unexplored, taking the form of real biological “dark matter”. Paradoxically, this little-studied region may contain genes, interactions, and network modules crucial to understanding many complex diseases, including those for which current therapeutic strategies are ineffective. The absence of data on these components limits not only the discovery of new biomarkers but also the ability to stratify patients and predict individual responses to treatments.

## 3. Limitations of Reductionist Approaches

Despite technological advances and the enormous availability of high-resolution biological data, a deep cultural and linguistic gap persists between data scientists—particularly physicists, mathematicians and computer scientists—and clinical clinicians who work daily in care practice [20]. These two worlds adopt different cognitive paradigms, methodologies and objectives: on the one hand, abstraction, modeling and quantitative analysis; on the other hand, clinical reasoning based on experience, patient context and uncertainty management. The absence of a shared language makes it difficult to translate computational results into operational clinical decisions. The advent of omics technologies—genomics, transcriptomics, proteomics and metabolomics—has further widened this gap. The ability to generate large volumes of data has grown at a much faster rate than our ability to interpret it in a biologically and clinically meaningful way. The increase in the amount of data does not automatically translate into an increase in useful knowledge, especially in the absence of a solid theoretical framework to guide its interpretation [15]. Without adequate conceptual models, data risks remain mere statistical correlations with no causal meaning.

In the clinical setting, this criticality is particularly evident in precision oncology. Molecular diagnostics laboratories are producing increasingly detailed reports containing lists of mutations, changes in the number of copies, transcriptomic signatures and potential therapeutic targets. However, such relationships are often structured according to technical and molecular logics that do not coincide with the clinician’s decision-making process [34]. The oncologist or general practitioner is thus faced with complex, sometimes ambiguous information that is difficult to integrate with the patient’s overall clinical picture, comorbidities, guidelines and therapeutic options available. This disconnect produces an operational paradox: while precision medicine promises increasingly personalized treatments, the complexity of information can translate into a reduction in its actual applicability. In the absence of shared interpretative tools, the risk is that clinical decisions remain anchored to traditional schemes, ignoring the potential added value of omics data, or that excessive weight is placed on weak or not clinically validated molecular evidence. Bridging this gap requires more than just technological solutions. A cultural change is needed that favors interdisciplinary training, the co-creation of interpretative models and the development of cognitive interfaces designed for the clinician. Data scientists must incorporate principles of pathophysiology, clinical relevance, and biological causation, while clinicians must be empowered to understand the limitations, assumptions, and uncertainty of computational models. Only through this integration will it be possible to transform the abundance of data into actionable knowledge and make data medicine a truly useful tool for clinical practice [20].

### 3.1. Clinical Implementation Challenges

In the era of precision oncology, the integration of complex molecular data into routine clinical practice represents both a major opportunity and a significant challenge. Although Molecular Tumor Boards (MTBs) were introduced with the aim of bridging the gap between molecular data complexity and clinical decision-making, their real impact on the therapeutic management of cancer patients remains, to date, relatively limited. Several observational studies show that only a small percentage of patients discussed in MTBs actually receive treatment based on the recommendations that emerged, often less than 15–20% [37,38]. This highlights a significant discrepancy between the theoretical potential of precision medicine and its practical application in the real world. The causes of this limited implementation are many and structural. Firstly, a significant portion of patients undergo molecular profiling at too advanced stages of the disease, when clinical conditions no longer allow access to experimental or innovative therapies. In these cases, even when an MTB identifies potentially “actionable” alterations, the patient is no longer eligible for targeted treatments or clinical trials. Added to this are the individual preferences of patients, who sometimes reject off-label or experimental therapies due to uncertainty about benefits, potential side effects or impact on quality of life. A further critical factor is the difficulty of accessing recommended drugs. Many of the therapeutic options suggested by MTBs concern molecules not yet approved for that specific indication, available only within clinical trials or through compassionate use programs. The availability of trials is highly heterogeneous across the territory and often limited to highly specialized centers, creating inequalities of access. In addition, regulatory and bureaucratic constraints further slow down the implementation of MTB decisions, reducing their operational effectiveness.

Alongside the clinical and organizational aspects, significant issues of economic sustainability emerge. Although molecular diagnostics represent a tiny fraction of overall healthcare costs—estimated at around 0.3% of drug and hospitalization expenses—Next-Generation Sequencing (NGS) tests still pose questions in terms of reimbursement, standardization and cost-effectiveness [38]. In many healthcare systems, access to advanced genomic testing is uneven and depends on pilot projects, research funds or local resources, making large-scale sustainable planning difficult. Even more complex is the issue of access to off-label or high-cost drugs suggested by MTBs. Yet, when rational biological evidence exists, reimbursement of such therapies is often uncertain or denied, limiting the effective translation of molecular recommendations into therapeutic interventions. This generates a structural tension between scientific innovation and the sustainability of health systems [39]. Overall, MTBs represent a fundamental but still incomplete tool in precision medicine. Their limited impact does not stem from a conceptual failure but from clinical, organizational, regulatory, and economic constraints that reduce their effectiveness. Overcoming these barriers requires earlier integration of molecular testing into the care pathway, better access to innovative drugs, and systematic evaluation of the clinical and economic value of personalized strategies so that the potential of MTBs can translate into concrete and sustainable benefits for more patients [37,39].

### 3.2. More Data Does Not Mean More Knowledge

The contemporary biomedical landscape is characterized by an “omics revolution” that has fueled a widespread sense of epistemic optimism. This perspective frequently equates the exponential accumulation of high-dimensional data with the linear growth of biological knowledge, assuming that a complete understanding of the human organism is merely a matter of increasing computational scale. However, this review argues that such “naïve dataism” confuses informative potential with clinical significance. In the absence of robust theoretical frameworks and explicit biological hypotheses, the current abundance of information risks amplifying uncertainty rather than reducing it, transforming complexity into an interpretative abyss. Data-driven medicine currently faces significant methodological hurdles, most notably the “curse of dimensionality.” When thousands of variables are analyzed across limited patient cohorts, the signal-to-noise ratio deteriorates, leading to the identification of spurious correlations—the so-called “Texas Sniper Fallacy”—where random statistical clusters are mistaken for mechanistic biomarkers. This methodological limitation underscores the urgent need for more robust and interpretable analytical frameworks capable of distinguishing true biological signals from noise. The core contribution of this work is to propose network-based models not merely as computational tools but as a shared, interpretable language capable of bridging the cultural and methodological divide between clinicians and data analysts. By integrating multidisciplinary expertise through the structural logic of network theory, we can move from a purely quantitative “big data” approach to a systems-based paradigm. Ultimately, the evolution of precision medicine depends on this synergistic integration, where numerical precision and medical reasoning act in concert to reconstruct the patient’s complexity into actionable clinical wisdom.

### 3.3. A Strategic Alliance: The Role of Molecular Tumor Boards (MTBs)

In the contemporary scientific landscape, the “omics revolution” has created a paradox: we are rich in data but poor in actionable clinical information. This difficulty stems from a profound epistemological divide between “number scientists”—who seek universal mathematical laws akin to Newtonian physics—and “life scientists,” who view biology as an idiographic discipline defined by historical contingency, diversity, and evolution. While the former often mistake data accumulation for biological understanding, the latter frequently lack the formal tools to navigate the overwhelming complexity of high-dimensional datasets. This fracture is rooted in the 17th-century triumph of mathematical abstraction, which successfully modeled the inanimate world but struggles with the nonlinear, adaptive nature of living systems. The failure of traditional reductionism—exemplified by the transition from the “magic bullet” theory to the recognition of multifactorial diseases—demonstrates that knowing the genetic alphabet is not equivalent to understanding the biological narrative. The central thesis of this review is that neither mathematics nor biology can solve this difficulty in isolation. We propose that networks serve as the essential cultural bridge, moving toward a transdisciplinary alliance. By sacrificing raw geographic fidelity for functional clarity—much like a transit map—network theory provides the shared language necessary to transform numerical precision into clinical wisdom, ensuring that algorithms serve as tools rather than tyrants.

**Figure 3:**
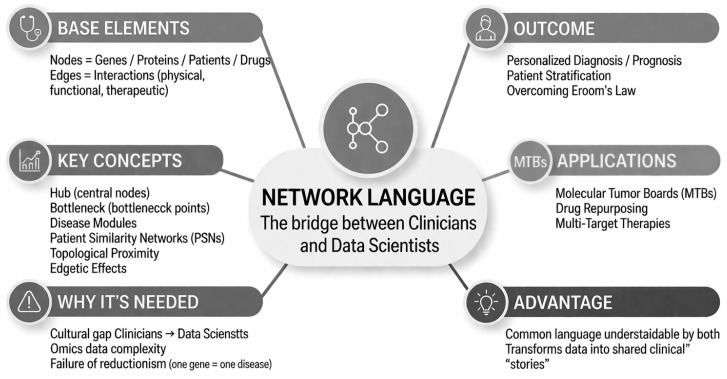
Conceptual map of the network language paradigm in precision medicine.

The current inability to obtain useful information stems from the fact that doctors and data analysts continue to work in silos. Analysts produce elegant but biologically irrelevant models, while doctors often drown in a mass of data that they do not know how to interpret quantitatively [40]. The solution lies in network-based precision medicine, which is not just a technique but a common language [8]. Network science offers a vocabulary of “computable metaphors” (community, centrality, connectors) that have a technical meaning for the analyst and a functional meaning for the physician [8]. When an analyst speaks of a “community” in a graph, the physician interprets it as a functional module, i.e., a group of molecules that cooperate for a specific cellular function [8]. A conceptual map is depicted in Figure 3.

This collaboration requires the creation of interdisciplinary platforms such as Molecular Tumor Boards (MTBs), where experts in pathology, oncology, bioinformatics and genetics dialogue to translate complex molecular data into personalized clinical choices [41,42,43]. In these forums, the decision is not entrusted to a “black box” algorithm but emerges from a reasoned interpretation of the data filtered through clinical experience [41,44].

For an effective implementation of genomics-driven precision medicine, Molecular Tumor Boards (MTBs) are critical tools. MTBs are multidisciplinary teams composed of oncologists, molecular pathologists, bioinformaticians, geneticists and other experts whose main task is to provide genomically informed clinical recommendations for cases with complex alterations. The recommendations are based on a multidisciplinary assessment and a case-by-case review of the literature, also taking into account patient-specific factors (such as health status and preferences). The molecular report is just the beginning, and MTBs are tasked with bridging the gap between the content of the report and the appropriate clinical decision [41,44].

The integration of NBPM into clinical practice finds its main operational arm in MTBs, multidisciplinary commissions created to face the challenge of precision medicine in an era dominated by complex data [41,42]. These forums are not limited to a simple reading of genomic reports but act as a real interpersonal and computational platform to translate interactome perturbations into personalized therapeutic decisions [43]. The starting point of this process lies in the awareness that cancer is not a disease defined by a single mutation but an emergent phenomenon that derives from the malfunction of entire disease modules within the network of protein interactions [36,45]. While the traditional approach seeks a “magic bullet” to hit a single gene, MTBs adopt a systemic view, recognizing that tumor robustness often lies in the functional redundancy of the network, making it necessary to hit multiple critical nodes simultaneously [46]. In this context, network-based precision medicine provides MTBs with topological tools to identify not only the most frequently mutated genes but also the so-called bottleneck or broker nodes, which, although not mutated, mediate the flow of pathological information between different functional modules and therefore represent hidden therapeutic targets [28,45].

Practical implementation takes place through a workflow that begins with the integration of multi-omics data (genomics, transcriptomics, proteomics) with the patient’s clinical profile. Bioinformatics experts within MTBs use algorithms such as DIAMOnD to map the patient’s “seed” genes to the human interactome, expanding the search to the most significant network neighborhood to identify the specific module that drives tumor progression in that individual [36,45,47]. This process makes it possible to generate the patient’s reticulotype, i.e., a personalized map of molecular interactions that takes into account the patient’s genetic and environmental uniqueness, overcoming the abstraction of the “average patient” [45,48].

Network-based precision medicine also enhances the ability of MTBs to suggest drug repurposing and rational polypharmacology [49]. By analyzing the network proximity between the target of an already approved drug and the patient’s disease form, MTBs can recommend off-label therapies with a solid mechanistic basis, reducing the risk of failure related to simple statistical correlations [45]. The multidisciplinary discussion also allows for evaluating the edgetic effects, i.e., how a mutation does not eliminate an entire protein but only alters a specific interaction, allowing MTBs to choose drugs that selectively correct the defective “wiring” without destroying healthy cellular functions [50,51].

Recent advances in network-based precision medicine enable systematic drug repurposing by quantifying the topological proximity between disease modules and drug targets within the human interactome. In Alzheimer’s disease, a stage-specific network proximity framework identified FDA-approved drugs whose targets closely align with molecular modules corresponding to mild cognitive impairment, early, and late AD. Integration with single-cell transcriptomics suggested cell-type-specific mechanisms, and selected compounds were experimentally validated for neuroprotective effects, highlighting the ability to capture temporal and cellular heterogeneity in neurodegeneration [52]. Similarly, a cardiovascular drug repurposing atlas applied network proximity to map ~984 approved drugs onto 23 cardiovascular disease modules. Predictions were validated using population-scale clinical datasets, confirming that non-cardiovascular drugs modulate disease risk in ways consistent with their interactome proximity, and mechanistic experiments supported these associations [53]. Collectively, these studies illustrate the versatility of network proximity as a mechanism-informed framework to prioritize repurposing candidates across diverse disease contexts.

Finally, MTBs act as the cultural mediator needed to overcome the complex data divide, i.e., the gap between number scientists and life scientists. Through the language of networks, abstract mathematical concepts such as “centrality” or “communities” are translated into clinical terms of “essentiality” and “functional cooperation”, allowing the clinician to maintain control over the final decision [8]. This ongoing dialogue ensures that each recommendation is not only statistically significant but also biologically plausible, avoiding the traps of algorithmic overfitting and ensuring that technology remains at the service of patient care in its entirety.

## 4. Principles of Network Medicine

To bridge this cultural gap, the proposal of network-based precision medicine emerges [54]. Network-based precision medicine is not just a technique but a common language based on graph theory, capable of translating biological concepts—such as cooperation, influence, and mediation—into computable mathematical patterns such as community, centrality, and connectors [55,56,57].

In this context, the doctor must not become a mathematician, nor the data analyst a biologist. Instead, the creation of interdisciplinary platforms, such as Molecular Tumor Boards (MTBs), allows experts in pathology, oncology, bioinformatics and genetics to dialogue on an equal footing. In these forums, the medical decision is not entrusted to a “black box” algorithm but emerges from a collective and reasoned interpretation of the molecular data filtered through clinical experience.

Furthermore—and no less important—the language of networks allows us to build “stories”, i.e., interpretations of biomedical big data that can be understood both in clinical and algorithmic terms, thus allowing direct doctor/data analyst dialogue and overcoming the cultural gap that divides them [27,54]. The simple concept of a network therefore becomes a tool for formulating clinical hypotheses on the causes of diseases and verifying them using data through dedicated algorithms. Conversely, patterns and structures found in the data using network-based precision medicine techniques can be presented to medical specialists to assess their biological plausibility and therapeutic potential.

**Figure 4:**
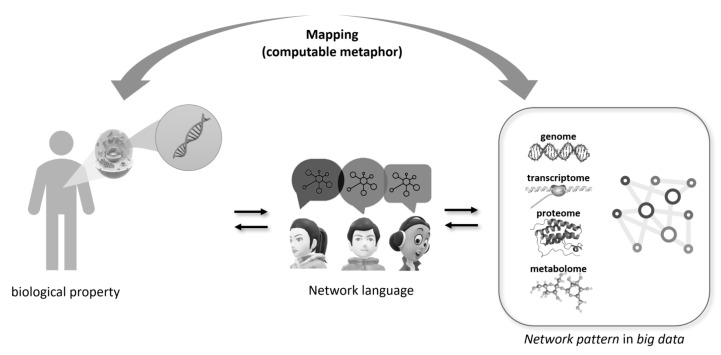
Conceptual framework illustrating the use of network-based representations as a computable metaphor linking biological properties to large-scale omics data. Patient-specific biological features (**left**) are translated into a shared “network language” that facilitates communication between clinicians and data analysts (**center**), enabling bidirectional interpretation. This mapping allows complex multi-omics layers (genome, transcriptome, proteome, metabolome) to be integrated into coherent network patterns (**right**), supporting more interpretable and clinically meaningful insights.

The crucial point is that the network functions as a calculable metaphor and not as an essential reality of a Platonic type. The web is not the “mathematics of life” but a way of representing data that makes sense both for those who analyze it and for those who interpret the results [54]. Through the analysis of networks of relationships, it is possible to make diagnoses, identify new drugs, find computational biomarkers, and estimate risk factors for complex diseases, such as cancer, cardiovascular diseases, autoimmune diseases (e.g., multiple sclerosis) and diabetes [27,54]. This opens a glimmer of hope that allows us to envision a future of real collaboration for interdisciplinary and collaborative medicine, capable of facing the formidable challenges posed by complex pathologies and biomedical big data. The conceptual framework of network-based precision medicine is illustrated in Figure 4.

### 4.1. Nodes, Arcs, and Structure

A network is an abstract representation composed of nodes and edges (or links) that model the relationships between entities. In biomedicine, nodes can represent different biological entities such as genes, proteins, metabolites, drugs, or patients, while arcs encode the interactions between these entities. These interactions can be physical, such as protein–protein bonds; functional, such as relationships between a gene and the phenotype; or therapeutic, such as the link between a drug and its molecular target [27,54,58].

Arches can take on different characteristics that define their meaning and usefulness. They can be directed, indicating the direction of the flow of information (for example, one gene that regulates the expression of another), or weighted, conveying the strength, probability or quality of the connection [59]. The use of weighted arcs is fundamental to analyzing quantitative biological networks, where the strength of molecular interactions influences the stability of the system and the therapeutic efficacy of any interventions.

Biological networks often have particular topological characteristics that distinguish them from simple random networks. One of the most studied properties is scale-free topology, in which the distribution of the degrees of the nodes follows a power law. In these networks, a small number of nodes, called hubs, have a very large number of connections, while most nodes have few connections [60]. Hubs play a critical role in maintaining network cohesion and transmitting biological signals. The presence of hubs gives the system robustness against random failures, since the random removal of nodes has little probability of affecting a hub but makes the network extremely vulnerable to targeted disruptions on the hubs themselves. This feature has relevant implications for medicine: for example, hub genes in genetic networks may represent potential high-influence therapeutic targets, while hub proteins in protein networks may be critical for cell survival [27,61].

In addition to scale-free topology, biological networks can exhibit other significant structural properties, such as high clustering, modularity, and the presence of recurring network patterns. High clustering indicates that nodes tend to form strongly connected subgroups, which often correspond to specific biological functions, such as a protein complex or metabolic pathway. Modularity allows for identifying functional “communities” in the network, offering insights into the hierarchical organization of biological processes and the development of targeted therapeutic approaches [58,62].

Networks also find application in patient medicine, where nodes can represent individuals and arcs can represent clinical or genetic relationships. In this context, network-based precision medicine allows us to study how genetic mutations, drug interactions and environmental risk factors combine to generate complex phenotypes and multifactorial diseases [27,54]. For example, patient–patient networks built on molecular similarities can help stratify patients into clinically relevant subgroups, while drug-target networks allow the identification of new pharmacological repositioning strategies [63].

In summary, biological networks are not simply a mathematical tool but a common language that allows biological complexity to be translated into quantitative and analyzable terms. Understanding the structure, function and dynamics of networks thus provides valuable information for diagnosis, prognosis and the discovery of new treatments, paving the way for interdisciplinary and data-driven medicine that overcomes the limitations of traditional reductionist analysis [27,54,58].

### 4.2. Modules, Hubs and Bottlenecks

Topological analysis of biological networks is one of the fundamental tools of network-based precision medicine, as it allows us to identify key nodes and subnetworks through quantitative metrics that describe the position and role of each node within the network. One of the simplest and most intuitive measures is degree centrality, which quantifies the number of connections to a node. In biological networks, high-grade nodes, called hubs, often correspond to essential genes, critical proteins, or strategic drug targets, the perturbation of which can have significant systemic effects [60,61,64]. Identifying hubs is crucial not only to understand the organization of the network, but also to identify possible therapeutic targets that can influence a large number of biological processes.

Another central measure is betweenness centrality, which assesses the importance of a node as a bridge within the network. This measure quantifies how many times a node is on the shortest path between other pairs of nodes, highlighting so-called bottlenecks. Nodes with high betweenness centrality play a strategic role in controlling the flow of information and coordinating the interaction between different biological modules. In pathological contexts, bottlenecks can correspond to critical points in metabolic pathways or regulatory signals, the perturbation of which can generate cascading effects that amplify the disease [27,65].

In addition to centrality, a fundamental property of biological networks is modularity, which reflects the tendency of nodes to group into subnets or modules with higher internal connection density than links to the rest of the network. Biological modules often correspond to protein complexes, metabolic pathways, or functionally coherent cellular processes. The identification of the modules allows for simplifying the complexity of the network and recognizing emerging functional patterns that would not be evident from the analysis of the individual nodes [62,66,67].

In the context of network-based precision medicine, a central concept is that of the disease module. A disease module is a connected subgraph of the biological network that includes the molecular determinants associated with a specific disease, such as genes, proteins, or pathological pathways. The hypothesis behind this concept is that the components of a pathology tend to localize in a specific vicinity of the interactome, rather than being randomly distributed throughout the network [27,64]. This spatial localization in the network can be exploited to predict candidate genes for diseases, identify new biomarkers or suggest targeted therapeutic strategies, based on the concept of topological proximity in the network [36].

The combined analysis of hubs, bottlenecks and modules allows us to build an integrated disease map in which each disease is represented by a functional area in the interactor, with critical nodes and clearly identified strategic connections. This approach allows us to study complex diseases in a systemic context, to predict comorbidities based on the intersection of disease modules, and to better understand responses to multiple pharmacological treatments [27,36,54].

**Figure 5:**
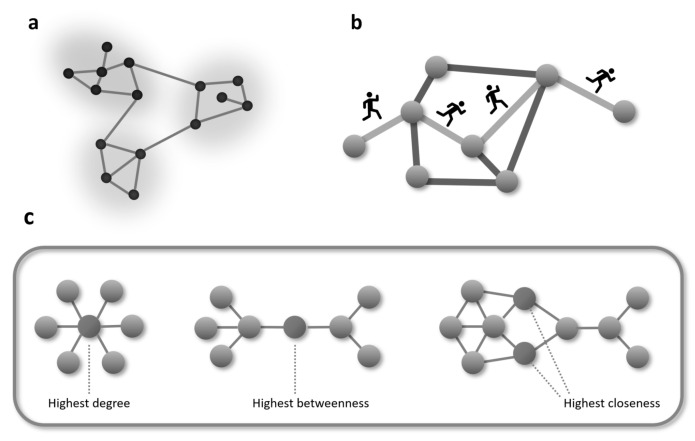
The figure illustrates several network patterns of relevance to precision medicine: (**a**) network modules, (**b**) the length of a path within a network, which is useful for defining distances between nodes, and (**c**) various network centrality measures.

In summary, topological analysis of biological networks provides powerful quantitative tools for understanding the organization of complex systems, identifying critical nodes and subnetworks, and linking the network structure to biological and pathological phenomena, laying the foundations for network-based medicine capable of integrating molecular, clinical, and pharmacological data into a coherent and interpretable framework (see Figure 5).

## 5. Clinical Applications

Cancer is a highly heterogeneous disease, characterized by numerous genetic, epigenetic and regulatory abnormalities that alter the molecular networks within the cell [54,68]. The complexity of molecular interactions makes it difficult to identify key determinants of disease using traditional single-gene approaches. Network-based precision medicine (NM) offers a systemic approach, allowing us to reconstruct cancer-specific networks by integrating transcriptomics, proteomics and interactome data, i.e., the overall map of molecular interactions between genes, proteins and metabolites [27,36]. Only differentially expressed molecule-sensitive interactions between tumor samples and normal tissues are included, improving the specificity of the analysis and reducing biological noise [69].

One of the fundamental principles of network-based precision medicine applied to cancer is guilt-by-association: genes that show high perturbations in expression (measured, for example, by log2 fold-change) and strong interactions with other genes that are also perturbed are assigned a higher score. This allows us to prioritize the “cancer genes” within the network and to identify critical nodes that may play a central role in tumor development and disease progression [27,54,69].

The main applications of this approach are divided into several areas:

1. Diagnosis (Discrimination): Gene analysis via the network makes it possible to discriminate between tumor samples and normal tissues with greater precision than methods based on single genes. Tumor networks identify coordinated expression patterns and functional subnetworks that represent more robust molecular signatures, improving sensitivity and diagnostic specificity [69,70].
2. Prognosis (Survival): Genes prioritized through network analysis can be integrated into multivariate Cox regression models to predict patient survival times. Recent studies have shown that the inclusion of network information improves patient stratification capacity, identifying high-risk subgroups that could benefit from more aggressive or targeted treatments [54,71].
3. Edge Perturbations: The analysis of edge perturbations, which evaluates alterations of specific interactions rather than individual nodes, allows the identification of molecular biomarkers and potential therapeutic targets. Edge perturbations consider that cancer does not always result from the loss or overactivation of a single gene but from the dysregulation of critical connections between molecules, which alter network pathways and modules [72,73]. This approach allows for more targeted targets to be discovered, which may not emerge from traditional analyses based on individual expression levels.

In summary, network-based precision medicine applied to cancer integrates molecular, topological and functional information, allowing us to build a systemic map of the disease. The analysis of critical nodes and connections provides tools for early diagnosis, personalized prognosis and discovery of therapeutic targets, transforming complex data into clinically useful and interpretable information [27,36,54].

### Drug Repurposing and Multi-Target Therapies

Network-based precision medicine (NM) represents an extremely powerful approach for drug repurposing, a process that aims to identify new therapeutic indications for already approved drugs, reducing time and costs compared to traditional development [27,74]. The central idea is that the position of a drug’s targets in the biological web, relative to the genes or proteins associated with a disease, can predict its therapeutic potential. In particular, a significantly short topological distance between drug targets and disease genes suggests that the drug can effectively influence underlying disease processes [36,63].

A fundamental aspect of the network-based precision medicine approach is the consideration of the regulatory direction in biological networks. In gene regulation or signaling networks, some nodes act as upstream, while others act as downstream. The repositioning takes this direction into account, favoring drugs whose targets are located upstream of the set of genes associated with the disease, so that the pharmaceutical intervention can effectively modulate the entire pathological cascade [54,63]. This approach reduces the risk of a drug acting on downstream nodes, which could have limited or even counterproductive effects.

A further advantage of network-based precision medicine analysis is the ability to assess potential toxicity on non-pathological tissues. By comparing the topological distance between drug targets and disease-associated genes versus the distance from genes expressed in normal tissues, potential side effects can be predicted and drugs with a more favorable safety profile can be selected [74,75]. This strategy allows efficacy and safety information to be integrated into a single, network-based quantitative model, improving the selection of drug candidates to be tested in clinical trials.

Numerous concrete examples show the effectiveness of this approach. Drugs such as lenalidomide and thalidomide, originally developed for other indications, have been identified through network-based precision medicine as candidates for the treatment of several forms of cancer, including multiple myeloma and some solid neoplasms. These findings derive from the analysis of their position in the network and distance from disease modules, which indicate a regulatory action consistent with altered pathways in tumors [27,63].

In addition to the drugs mentioned, network-based precision medicine has made it possible to identify numerous other candidates for repositioning in cardiovascular, neurodegenerative and autoimmune diseases, confirming the generalizability of the method [74,75]. The network-based approach allows us not only to discover new uses of existing drugs but also to propose synergistic drug combinations, selecting pairs of drugs whose targets complement each other in the modulation of disease modules while reducing the risk of toxicity.

In summary, network-based precision medicine provides a systemic and quantitative framework for drug repurposing, integrating information on interactome, differential gene expression, network topology, and regulatory signal direction. This approach not only accelerates the discovery of new therapeutic indications but also increases the likelihood of clinical efficacy and minimizes the risk of side effects, representing a modern and powerful strategy to address complex diseases [27,63,74].

## 6. Future Directions

Network-based precision medicine today represents a fundamental strategy for managing and interpreting large heterogeneous datasets, translating complex information into exploitable biological and therapeutic knowledge [54,76]. The discipline directly supports precision medicine, aiming at a mechanistic stratification of patients that goes beyond simple clinical categorization based on observable phenotypes [20]. Despite advances, network-based precision medicine faces significant challenges, including the incompleteness of omics and interactome data, which can limit the ability to fully map biological networks and accurately predict the impact of molecular perturbations [77].

The network-based precision medicine paradigm emerges as a response to the biological and clinical complexity that has made the traditional approach of precision medicine insufficient. The mere availability of omics and clinical data does not automatically guarantee useful knowledge: the complexity of molecular interactions, the fragmentation of biological maps, the limitations of reductionist models and the cultural differences between data scientists and clinicians constitute significant obstacles [4,60,78]. Network-based precision medicine proposes a common language based on graph theory, systems biology and clinical interpretation, making it possible to integrate molecular, phenotypic and clinical data into a coherent framework.

In this context, complex diseases are no longer considered as the result of the alteration of single genes but as perturbations of functional modules within the interactome, allowing more precise diagnoses, identification of innovative biomarkers, and development of multi-target therapeutic strategies [36,63,64,79,80]. Operational tools such as Molecular Tumor Boards concretely show the value of an interdisciplinary approach: they integrate complex molecular data with clinical experience, transforming computational analyses into personalized and biologically plausible therapeutic decisions [81,82].

From an educational point of view, introducing network teaching in university courses has become essential in training professionals capable of dealing with complex systems in the life sciences [59,83]. Networks offer a common language that represents genes, proteins, metabolites, drugs and patients as nodes interconnected by functional relationships, providing a systemic view of biological phenomena and overcoming traditional reductionism [14,60]. In this way, students learn to integrate quantitative skills and biological knowledge, bridging the cultural gap between data scientists and clinical biologists. Networks help transform large amounts of heterogeneous data into interpretable structures, developing critical capabilities to distinguish spurious correlations from biologically meaningful relationships.

Network-based precision medicine is already successfully applied in advanced clinical settings such as Molecular Tumor Boards, where network models guide diagnosis, prognosis and drug repositioning, demonstrating the concreteness of the concept of network-based personalized medicine. Integrating these teachings into universities means preparing a new generation of researchers and clinicians who can interpret complexity, collaborate effectively, and translate big data into clinically actionable knowledge.

In conclusion, the revolution in medicine will not be driven by algorithms alone but by the integration of these tools with clinical wisdom. Network-based precision medicine is the foundation of this new alliance, providing conceptual and computational tools to consider the patient as a whole and put data at the service of care, not vice versa. Only by recognizing the uniqueness of the living and adopting a systemic perspective will it be possible to transform big data into clinically relevant knowledge.

## Data Availability

No new data were created or analyzed in this study.
